# Gender differences among Indigenous Canadians experiencing homelessness and mental illness

**DOI:** 10.1186/s40359-019-0331-y

**Published:** 2019-08-28

**Authors:** Brittany Bingham, Akm Moniruzzaman, Michelle Patterson, Jitinder Sareen, Jino Distasio, John O’Neil, Julian M. Somers

**Affiliations:** 1 Somers Research Group, Faculty of Health Sciences, Simon Fraser University, Burnaby, BC Canada; 2 Psychology and Community Health Sciences, University of Manitoba, Winnipeg, MB Canada; 3 Geography, University of Winnipeg, Winnipeg, MB Canada; 4 Faculty of Health Sciences, Simon Fraser University, Burnaby, BC Canada

**Keywords:** Canada, Homelessness, Indigenous, Mental health, Substance use, Service use, Women, Gender differences

## Abstract

**Background:**

Indigenous people are over represented among homeless populations worldwide and the prevalence of Indigenous homelessness appears to be increasing in Canadian cities. Violence against Indigenous women in Canada has been widely publicized but has not informed the planning of housing interventions. Despite historical policies leading to disenfranchisement of Indigenous rights in gender-specific ways, little is known about contemporary differences in need between homeless Indigenous men and women. This study investigated mental health, substance use and service use among Indigenous people who met criteria for homelessness and mental illness, and hypothesized that, compared to men, women would have significantly higher rates of trauma, suicidality, substance dependence, and experiences of violence.

**Methods:**

This study was conducted using baseline (pre-randomization) data from a multi-site trial. Inclusion in the current analyses was restricted to participants who self-reported Indigenous ethnicity, and combined eligible participants from Vancouver, BC and Winnipeg, MB. Logistic regression analyses were used to model the independent associations between gender and outcome variables.

**Results:**

In multivariable regression models among Indigenous participants (*n* = 439), female gender was predictive of meeting criteria for PTSD, multiple mental disorders, current high suicidality and current substance dependence. Female gender was also significantly associated with reported physical (AOR: 1.52, 95% CI = 1.10–2.23) and sexual (AOR: 6.31, 95% CI = 2.78–14.31) violence.

**Conclusions:**

Our analyses of Indigenous men and women who are homeless illustrate the distinct legacy of colonization on the experiences of Indigenous women. Our findings are consistent with the widely documented violence against Indigenous women in Canada. Housing policies and services are urgently needed that take Indigenous historical contexts, trauma and gender into account.

**Trial registration:**

This trial has been registered with the International Standard Randomized Control Trial Number Register and assigned ISRCTN42520374; ISRCTN57595077; ISRCTN66721740.

## Background

Indigenous^1^ peoples are overrepresented among homeless populations worldwide and urban Indigenous homelessness is on the rise [1–4]. In Canada, Indigenous people are five times more prevalent among the homeless compared with the general population [1, 3]. Indigenous people account for 2.5% of Canada’s population but comprise 38% of the homeless in Vancouver, BC [5]. Recent estimates indicate that over 30% of the Canadian shelter population is Indigenous, a crisis that is rooted in historical determinants and systemic discrimination [3]. Longstanding structural inequities associated with colonization (e.g., the Indian Act, residential schools, marginalization and systemic racism, and dispossession of lands) are commonly noted as contributors to Indigenous homelessness [1–3, 6]. However, The effects of colonial policies, including disconnection from community, culture, and lands are experienced differently by Indigenous men and women, and these gendered perspectives have received little attention from researchers [2, 6].

Historically, Indigenous women played central and powerful roles in their communities as matriarchs and the custodians of cultural and tribal traditions [7]. Indigenous women have been subjected to coercive racialized policies intended to subjugate their traditional matriarchal roles and authority [8]. In 1850, The Canadian government introduced a definition of “Indigenous”, whereby women forfeited their Indigenous status when they married a non-Indigenous man, while Indigenous men retained their status regardless of spousal ethnicity [8]. Furthermore, Indigenous women were legally denied the right to any marital property upon divorce or separation with men retaining the right to the family home [9]. The right to property defined under the Indian Act, has had far reaching implications in the lives, mobility and safety of Indigenous women. A patriarchal social structure was imposed by European settlers, and over time the belief that women were inferior to men began to influence Indigenous communities as increasing numbers of Indigenous women were disenfranchised and compelled to raise their families without customary connections to tradition, culture or community. A gendered hierarchy was also integral to the operation of Indian residential schools and continues to influence the marginalization of Indigenous women [10]. Indigenous gendered health disparities must be framed by discussions of the distinct historical experiences of Indigenous men and women and the contemporary impact these factors have on Indigenous marginalization, street involvement, trauma and homelessness.

Menzies (2009) qualitatively examined the experiences of homeless Indigenous men and found that family disconnection, intergenerational trauma and violence were subjectively the key influences on their life trajectories [11]. Despite the shared historical traumas of Indigenous men and women, Indigenous women in Canada bear a disproportionate burden of disease, poverty, homelessness, violence and incarceration [12]. Violence toward Indigenous women in Canada has been identified as a national human rights crisis [13, 14]. There have been more than 160 cases of missing or murdered Indigenous women and girls in the province of British Columbia and 79 cases in Manitoba, most of which are unsolved [13, 15, 16]. Indigenous Canadians’ rates of violent victimization are more than double those of the non-Indigenous population and rates of sexual assault are nearly 3 times higher than for non-Indigenous Canadians [17]. Violence shapes the context within which Indigenous women access (or avoid) housing and health services, and contributes to their “invisibility” [18, 19]. Indigenous scholars have called for research examining how colonialism, racism and sexism determine health and healthcare access among Indigenous women [7, 12, 20]. Little is known about homelessness among Indigenous women and their experiences of violence, trauma, and coercion, which may shape their interactions with service providers.

Beyond ethnicity, additional barriers are experienced by women who are mothers struggling to maintain housing for their families [21]. Thurston, Turner and Bird (2016) discuss the role of domestic and interpersonal violence (IPV) as a key contributor to homelessness among Indigenous women, and the racialized barriers Indigenous women face when seeking support for IPV, including the absence of culturally appropriate supports for women and their children [6]. In addition, being female and Indigenous carries an increased risk of trauma (e.g., PTSD), often as a consequence of violent assault [14]. Recent research has discussed the crucial importance of expanding culturally relevant and safe services for Indigenous peoples’ as a whole [12]. However, few studies have investigated the relevance of cultural and gender-based differences within marginalized Indigenous groups [22].

Indigenous women are overrepresented among homeless women [3, 23–25], and are more likely than men to provide care for children or other family members while experiencing core housing need [3, 26]. No known research to date has quantified the impact that homelessness among Indigenous women has on the ability of their children and future generations to thrive. In 2016, 60% of children and youth in care in BC were Indigenous and this percentage has been increasing over time with Indigenous children 15 times more likely to be in care than non-Indigenous children [27]. Intergenerational trauma is a direct result of colonial child welfare policies from the 1960’s at the height of the residential school era, when provinces removed Indigenous children and placed them in foster care, most often with non-Indigenous families [27]. Indigenous women are five times more likely than non-Indigenous women to be single parents [7]. Research on marginalized women engaged in sex work in Vancouver found that over one-third had their children forcibly removed and that Indigenous women had a 66% greater odds of child apprehension compared to non-Indigenous women [28]. Some authors contend that continued apprehension of Indigenous children into government care is contemporary cultural genocide, evidenced by the fact that the number of children in care today exceeds the number at the height of residential schools [27, 29]. Little is known about the impact of historical and current child apprehensions on homeless Indigenous women’s levels of trauma and trust for government programs and services.

Several studies indicate that pathways to homelessness differ based on gender [30, 31]. For example, homeless women are more likely to be victims of violence compared to men and more likely to report family disruption or dysfunction while men are more likely to report loss of a job, mental illness, and substance use problems as precursors to homelessness [32]. Clark and Rich (2003) highlighted that homeless populations are diverse in ethnicity, gender and pathways to homeless and recommended matching housing interventions to the characteristics of the service users [33]. However, few studies have focused on the distinct needs of Indigenous women who are homeless, contributing to their ‘invisibility’ in the public policy realm [34].

Our study aimed to address this gap by investigating the relevance of gender in a large sample of Indigenous people who experienced homelessness and mental illness in two Canadian cities (Vancouver, British Columbia; Winnipeg, Manitoba). Our study focus was within Indigenous peoples, and not a comparison between people of differing ethnicities. We hypothesized, based on the forgoing historical and contemporary rationale, that compared to Indigenous men, Indigenous women would have significantly higher prevalence of current trauma, suicidality, substance dependence, and recent experiences involving violence and threats.

## Methods

This study is part of a 4-year research demonstration project, the At Home Study, which investigated the effectiveness of Housing First^2^ in five Canadian cities, with each site contributing a different emphasis specific to the local context. The Winnipeg site addressed the needs of Indigenous people, while Vancouver addressed addiction [35, 36]. The current study drew from baseline data from both Vancouver and Winnipeg due to the over-representation of Indigenous people among those who are homeless in both cities [35–37].

### Ethics statement and community engagement

Our multi-site randomized trial included people who met criteria for homelessness and mental illness. Community engagement processes were specific to each site. In Vancouver focus groups and advertised open meetings were conducted with people who had been or were homeless, convened in custody and community settings, and at an Indigenous support centre [36, 37]. Indigenous people and other community stakeholders were engaged in the development and implementation of this research. Investigators engaged organizations providing services to Indigenous people who were homeless or precariously housed. People with Indigenous ancestry were members of the investigative team in roles as field researchers, research coordinator, and co-investigators [37]. Winnipeg convened a Cultural Lens Committee comprised of elders and traditional teachers to provide guidance to the project. Indigenous teachings and practices were integrated into the interventions delivered in Winnipeg, and a Lived Experience Circle was created to ensure that Indigenous experiences were honoured and promoted through the research. Throughout the Winnipeg site, participants and persons with lived experience (PWLE) were involved extensively with multiple roles on the project such as representatives on an advisory committee, employed as research staff and assigned key roles working with service providers [37]. PWLE assisted in facilitating integrated knowledge exchange working with staff of the interventions and directly to bring participant perspectives into the research and interventions [38]. Details of the study protocols are published elsewhere [35, 36]. The parent trials are registered as follows: ISRCTN42520374; ISRCTN57595077; ISRCTN66721740. All variables were collected pre-randomization. Ethical review and approval was conducted by the Research Ethics Boards at Simon Fraser University, The University of British Columbia and the University of Manitoba with endorsement from the University of Winnipeg.

### Study sites

Vancouver has a large visible population of homeless and mentally ill individuals located within a central downtown neighborhood which is characterized by high crime rates and an open drug market [35]. The Downtown Eastside of Vancouver (DTES) includes a high number of single room occupancy (SRO) hotels which comprise the least expensive housing in the area and have been characterized as bed bug infested places of criminal predation [39–41]. SRO’s have become the only housing option for many low income individuals who depend on government assistance [39]. The greater Vancouver region is home to 11 First Nations communities neighboring or within the urban center [42]. British Columbia is home to 203 distinct First Nations province-wide, many of which come to the city center of Vancouver to receive health services. Métis Nation BC represents 38 distinct Métis communities and there are nearly 90,000 self-identified and 18,000 provincially registered Métis people in BC [43]. Vancouver is a city characterized by extremely high property prices and lack of affordable housing as well as the ‘deinstitutionalization’ of those with mental illness to community setting with insufficient supports [35].

The city of Winnipeg was selected as a site for the At Home/Chez Soi study due to an overrepresentation of Indigenous people in the city who were homeless and living with mental illness. Winnipeg is a prairie city with a population of approximately 730,000 and home to the country’s largest urban Indigenous population with over 72,000 identifying in the last census [38]. The growing urban Indigenous population is younger than the general population, with Indigenous children representing closer to 30% of the total Indigenous population in Winnipeg [38]. It has been estimated that more than 80% of Winnipeg’s homeless have originated from Indigenous communities. Winnipeg has had a vacancy rate below 2% since 2001and long waitlists persist for those seeking affordable shelter. Similar to Vancouver, rental housing supply in Winnipeg is located mostly in the inner city where often housing is older or in need of repair [38].

### Participants

Participants were recruited through referral from over 40 agencies in Vancouver, and 50 agencies in Winnipeg, including: homeless shelters, drop-in centers, homeless outreach-teams, hospitals, community mental health teams, and criminal justice programs. Eligibility criteria included: being a legal adult (19 years or older), current mental disorder, and being absolutely or precariously housed [35, 44]. Absolute homelessness was defined as living in a shelter or on the streets for a minimum of two weeks in the past year [36]. Precarious housing was defined as living in a rooming house, hotel or transitional housing, individuals must also have experienced at least two episodes of absolute homelessness in the past year with one episode lasting for at least 4 weeks. [35, 36, 45]. For inclusion, participants must have experienced two or more episodes of absolute homelessness in the past year or one episode lasting at least one month in the past year [35].

### Data collection

An initial face-to-face interview was conducted to screen for eligibility. Trained research staff conducted interviews and obtained written informed consent from all participants [36, 46]. Enrolled participants provided socio-demographic information and details concerning homelessness, mental illness, substance use, physical health, service use, and quality of life [36]. The following standard questionnaires were administered: Health service access [36]; Community integration scale; Health related quality of life (EuroQol 5D) [47–49]; Global Appraisal of Individual Needs (GAIN-SPS), Substance Problem Scale [50]; SF-12 Health Survey [51]; Quality of Life Index, 20-item [52]; Mini International Neuropsychiatric Interview (MINI) [53]; Social support items and food security; Health, social, and justice use inventory [36]; and Multnomah Community Ability Scale (MCAS) [54]; Recovery Assessment Scale, 22-item [47, 48] and Adverse Childhood Experiences Questionnaire [36]. Detailed descriptions of the instruments included in the questionnaire are described in previous protocol papers [36]. Participants received a cash honorarium of $30 upon completion of the baseline interview and $20 for each subsequent interview.

### Variables

Independent variables included gender, ethnicity, and level of need. *Gender* was categorized based on the question, “What is your gender? Do you identify as: male, female, transgender, transsexual, other, declined.” *Indigenous ethnicity* was self-reported and did not include participants who indicated they were part-Indigenous or who self-identified as “Mixed/Other”. Participants who self-identified as Indigenous and were asked to specify: Inuit, First Nations Status, Indigenous from outside Canada, Métis, First Nations Non-Status, and other. Participants’ *level of need* was categorized “high needs” (HN), based on the MCAS score of 62 or lower or current bipolar or psychotic disorder; as well as one of the following: legal involvement in the past year, substance dependence in the past month, and two or more hospitalizations for mental illness in the past 5 years. All other eligible participants were categorized as “moderate needs” (MN) [35].

Dependent variables included posttraumatic stress disorder (PTSD), multiple mental disorders, suicidality, substance and alcohol dependence and experiences of violence.

Participants’ *mental illness* was categorized as severe or less severe. The severe cluster of mental disorders includes at least one of current (i.e. past month) psychotic disorder, mood disorder with psychotic features, and hypomanic or manic episode, as identified through the MINI. The less severe cluster includes at least one of current major depressive episode, panic disorder and posttraumatic stress disorder (PTSD) [35]. *Substance dependence* was also identified using the MINI and GAIN-SPS. Frequency of use included all illicit drugs (i.e. not alcohol). *Infectious disease* was based on a positive self-report diagnosis of HIV, Hepatitis B, or Hepatitis C [35]. *Experiences of violence in the past 6 months* were identified by asking participants “During the past 6 months, did anyone take or try to take something from you by force or threat of force?”. Participants were also asked “During the past 6 months, did anyone threaten to hit or attack you, or threaten you with a weapon?” and “During the past 6 months, has anyone forced you or attempted to force you into any unwanted sexual activity, by threatening you, holding you down or hurting you in some way?” Participants who answered ‘yes’ were considered to have experienced violent victimization in this analysis.

Self-reported involvement with *health services* was collected for the past 6 months and included visiting a family doctor, psychiatrist, Emergency Room (ER), and transport by ambulance. Access to health care was categorized based on the questions, “Is there a place that you usually go to when you’re sick or in need of advice about your health?” and “In the past 6 months, was there ever a time when you needed health care but you did not receive it?” Criminal justice services included contact with the police that did not result in arrest and contact that resulted in arrest or being held in a police cell for less than 24 h.

### Statistical analysis

Pearson Chi-square tests or Fisher’s exact test were used to conduct comparisons of categorical variables between groups (Vancouver vs. Winnipeg participants; Indigenous vs. non-Indigenous participants and Indigenous female vs. Indigenous male participants). Comparisons of numeric variables (e.g., age at enrolment) between groups were conducted using the Student t- test and Wilcoxon’s rank-sum test as appropriate. Comparisons were conducted across socio-demographic variables, homelessness variables, mental disorder, substance use, health conditions, trauma, violence and service use. Univariate and multivariable logistic regression analyses were used to model the independent associations between gender and a series of a priori outcome variables. Outcome variables that were significant at the *p* < 0.05 level in univariate analysis were considered for inclusion in the multivariable logistic regression analyses. Each multivariable model included gender (primary independent variable) and the following controlling variables: age (continuous); gender (male, female); ethnicity (Aboriginal, White, other); need level (high, moderate); marital status (single, other); site (Vancouver, Winnipeg); education (completed high school, incomplete high school); have children (under age 18). Given the exploratory nature of our study we did not control for multiple comparison [55, 56]. Both unadjusted and adjusted odds ratios and 95% Confidence intervals (CI) are reported. SPSS Version 21 was used to conduct these analyses. Institutional review and ethics approval was provided by Simon Fraser University’s office of Research Ethics application number 2009 s0231.

## Results

### Sample characteristics

In Vancouver, 497 participants met eligibility criteria and 77 (16%) identified as Indigenous. Of the 513 eligible participants in Winnipeg, 362 (71%) identified as Indigenous. Selected baseline characteristics for the full sample (*n* = 1010) are summarized here and additional details have been published elsewhere [37]. Differences in the proportions of Indigenous and non-Indigenous people in each site were associated with further differences involving need level, gender, ethnicity, education, hospitalizations, arrests, housing status, mental illness severity, and suicidality (*p* < 0.05). In both Vancouver and Winnipeg, most participants were male: 359 (73%) and 326 (64%) respectively. Five participants self-identified as transgender and 2 participants identified as transsexual. Although the small number of transgender and transsexual does not enable separate analyses, it is worth noting that 5 of the 7 people who identified as transgender or transsexual also identified as Indigenous.

In total, 439 people (44%) from the combined sample identified as Indigenous. Most Indigenous participants were male (61%); categorized as moderate needs (59%); had not completed high school (75%); and reported a lifetime duration of homelessness greater than 3 years (52%). Compared to non-Indigenous participants, Indigenous participants were more likely to have children under the age of 18-years; report hospitalization for mental illness; and report lifetime durations of homelessness longer than three years.

Compared to Indigenous men, women were significantly more likely to be younger (*p* = 0.002); single (*p* = 0.03); and have minor-aged children (*p* < 0.001; see Table 1). Indigenous women were also more likely than Indigenous men to meet criteria for high levels of suicidality (*p* < .001); PTSD (p = 0.002); less severe mental disorder (*p* = 0.034); and two or more mental disorders (p = 0.002). Furthermore, Indigenous women were more likely than Indigenous men to report experiences of violence, including: forced or attempted unwanted sex (p < 0.001); attempts to take something by force or threat (*p* = 0.048); threats to hit or attack them (*p* = 0.039).

**Table 1:** Socio-demographic, mental health, substance use and service use characteristics for Vancouver and Winnipeg Indigenous Female and Male Home Study Participants (*N* = 439)

Variable	Indigenous Female (*N* = 169, 38.9%) *N* (%)	Indigenous Male (*N* = 265, 61.1%) *N* (%)	*P* value
Need Level			
High Need	74 (43.8)	104 (39.2)	0.348
Moderate Need	95 (56.2)	161 (60.8)	
Age at Enrollment			
Youth	25 (14.8)	28 (10.6)	**0.002**
25–44 Years	100 (59.2)	161 (60.8)	
44 Plus Years	44 (26.0)	76 (28.7)	
Education			
High School or Higher	42 (25.0)	67 (25.4)	**0.008**
Less than High School	126 (75.0)	197 (74.6)	
Marital Status			
Single (never married)	117 (69.2)	191 (72.6)	**0.030**
Married/Partner	18 (10.7)	11 (4.2)	
Separated/Widow/divorced	34 (20.1)	61 (23.2)	
Have Children (under 18)	111 (66.5)	111 (42.7)	**< 0.001**
Hospitalized for mental illness over 6 months in past 5 years	9 (5.4)	13 (5.0)	0.870
Hospitalized for mental illness over 2 times in the past 5 years	44 (26.7)	62 (23.6)	0.471
Arrested/Imprisoned/Probation/Community sanction in past 6 months	57 (33.9)	111 (41.9)	0.098
One or more nights in hospital, detox, shelter and jail in last 6 months	155 (91.7)	226 (85.3)	**0.046**
Length of Homelessness Lifetime			
1–3 years	81 (49.7)	123 (47.9)	0.714
3 years plus	82 (50.3)	134 (52.1)	
Length of homelessness lifetime			
12 months	33 (20.2)	56 (21.8)	0.833
13–60 months	72 (44.2)	106 (41.2)	
60 months plus	58 (35.6)	95 (37.0)	
Length of homelessness longest single period			
12 months	67 (41.6)	121 (47.8)	0.216
13–60 months	69 (42.9)	95 (37.5)	
60 months plus	25 (15.5)	37 (14.6)	
Age first homeless			
18 years or less	50 (29.8)	78 (29.8)	0.643
19–30 years	54 (32.1)	86 (32.8)	
31–40 years	30 (17.9)	56 (21.4)	
Over 40 years	34 (20.2)	42 (16.0)	
Housing Status			
Absolutely Homeless	111 (65.7)	196 (74.0)	0.064
Precariously Housed	58 (34.3)	69 (26.0)	
Mental illness & substance use			
Less severe mental illness	150 (88.8)	215 (81.1)	**0.034**
Multiple mental disorders (≥2)	126 (74.6)	160 (60.4)	**0.002**
Current PTSD	98 (58.0)	114 (43.0)	**0.002**
Current suicidality	160 (94.7)	216 (81.5)	**< 0.001**
Current alcohol dependence	111 (65.7)	176 (66.4)	0.875
Current substance dependence	108 (63.9)	130 (49.1)	**0.002**
Age first alcohol use			
> 14 yrs.	62 (38.5)	111 (42.7)	0.397
< 13 yrs.	99 (61.5)	149 (57.3)	
Age first drug use			
> 14 yrs.	73 (45.9)	124 (49.4)	0.491
< 13 yrs.	86 (54.1)	127 (50.6)	
GAIN-SPS score of substance use			
Less severe (0–3)	86 (55.1)	158 (63.2)	0.106
Severe [4, 5]	70 (44.9)	92 (36.8)	
Physical illness, service utilization and trauma			
Blood borne diseases	46 (27.5)	74 (28.2)	0.875
Two or more physical illness	159 (94.1)	225 (84.9)	**0.004**
Have a regular medical doctor	131 (77.5)	164 (61.9)	**0.001**
Place you usually go when you are sick or need advice about your health	146 (86.4)	222 (84.4)	0.572
Needed health care, but did not receive it in past 6 months	91 (54.5)	130 (49.8)	0.344
During past 6 m did anyone take or try to take something from you by force or threat of force?	78 (47.0)	97 (37.3)	**0.048**
During past 6 m did anyone threaten to hit or attack you, or threaten you with a weapon?	105 (63.6)	139 (53.5)	**0.039**
During past 6 m has anyone forced you or attempted to force you into any unwanted sexual activity, by threatening you, holding you down or hurting you in some way?	31 (18.7)	9 (3.5)	**< 0.001**

PTSD: Post-Traumatic Stress Disorder

GAIN-SPS: Global Assessment of Individual Need –Substance Problem Scale

Data in bold are outcome variables that were significant at the p < 0.05 level

Unadjusted (UOR) and adjusted odds ratios (AOR) and 95% confidence intervals included in the univariate and multivariable analyses are presented in Table 2. Results from multivariable logistic regression analyses confirm the findings of the bivariate comparisons and indicate that among the Indigenous participants, female gender was predictive of meeting criteria for PTSD (AOR: 1.81; 95% CI = 1.22–2.69); multiple mental disorders (AOR: 1.86; 95% CI = 1.20–2.86); current high suicidality (AOR: 3.97; 95% CI = 1.88–8.4); and current substance dependence (AOR: 1.91; 95% CI = 1.26–2.91). Female gender was also predictive of reports of violence such as, someone taking something from them by force or threat of force (AOR: 1.52, 95% CI = 1.10–2.23), someone threatening to hit or attack them or threatening them with a weapon in the past 6 months (AOR: 1.51, 95% CI = 1.00–2.27), and someone forcing or attempting to force sexual activity on them by threatening or holding them down or hurting them (AOR: 6.31, 95% CI = 2.78–14.31).

**Table 2:** Logistic Regression analysis to estimate the effect of gender on mental illness, substance use, trauma and violence among Vancouver and Winnipeg Indigenous ‘At home’ participants (N = 439)

Dependent variable	Unadjusted OR (95% CI)	P Value	Adjusted OR (95% CI)	*P* Value
Current PTSD	1.83 (1.24–2.70)	**0.002**	1.81 (1.22–2.69)	**0.003**
Multiple mental disorders (> 2)	1.92 (1.26–2.94)	**0.003**	1.86 (1.20–2.86)	**0.005**
Current suicidality	4.03 (1.93–8.45)	**< 0.001**	3.97 (1.88–8.4)	**< 0.001**
Current substance dependence	1.84 (1.24–2.73)	**0.003**	1.91 (1.26–2.91)	**0.002**
During past 6 m did anyone take or try to take something from you by force or threat of force?	1.49 (1.00–2.21)	**0.048**	1.52 (1.10–2.23)	**0.042**
During past 6 m did anyone threaten to hit or attack you, or threaten you with a weapon?	1.52 (1.02–2.27)	**0.039**	1.51 (1.00–2.27)	**0.046**
During past 6 m has anyone forced you or attempted to force you into any unwanted sexual activity, by threatening you, holding you down or hurting you in some way?	6.35 (2.94–13.74)	**< 0.001**	6.31 (2.78–14.31)	**< 0.001**

Controlled for age (continuous), need level (high, moderate), marital status (single, other), site (Vancouver, Winnipeg), education (high school or higher, less than high school), have children (under 18)

PTSD: Post-Traumatic Stress Disorder

Data in bold are outcome variables that were significant at the p < 0.05 level

## Discussion

The results of this study are consistent with the hypothesis that Indigenous women who become homeless are markedly afflicted by violent victimization, post-traumatic stress and suicidality. Indigenous women were 6 times more likely than Indigenous men to be victims of forced sexual violence, and were significantly more likely to exhibit symptoms of PTSD and suicidality. Our findings support the interpretation that Indigenous women continue to experience forms of violence, social exclusion, and marginalization. Women comprised a substantially higher percentage of our Indigenous sample (39%) compared to our non-Indigenous sample (26%). These findings can be meaningfully interpreted as a reflection of colonizing practices that sought to extinguish Indigenous women’s historic roles as matriarchs and custodians of cultural continuity. However, our research design does not address the causal role of these historical factors.

Our results highlight the importance of recognizing the distinct experiences of Indigenous women when providing housing and relevant support services, particularly those emphasizing culturally and trauma informed care. Consistent with other research [2, 17], our study found that Indigenous women experience violence at alarming rates. A 2009 Statistics Canada study reported that rates of violent victimization for Indigenous women (63% of females between the ages of 15 to 34) were three times higher than for non-Indigenous women [57, 58]. Homelessness literature has discussed differential impacts of sexual victimization among women compared to men in terms of increased psychopathology and legal involvement [32, 59]. Halseth discusses violence as a serious threat to the wellbeing of Indigenous women, perpetuating a gender specific form of marginalization established under colonization [7]. Support services for Indigenous women survivors of violence urgently need to be strengthened to reverse these historically rooted forces.

Indigenous women in this study were almost 2 times more likely than Indigenous men to be diagnosed with post-traumatic stress disorder (PTSD). PTSD is closely linked to historical and current experiences of violence experienced by Indigenous peoples. Research has documented that women are more likely to develop PTSD than men [60], and high rates of PTSD have been described among residential school survivors; however with known low rates of service engagement in Indigenous communities these may be underestimates [14].

Indigenous women in this study were close to 4 times more likely than Indigenous men to have high suicide risk or suicidality. Almost every Indigenous woman in this analysis actively thought about death and suicide with high suicidality among 95% of respondents. This shocking statistic is consistent with the sparse research that exists documenting the suicide crisis among Indigenous Canadians, particularly that Indigenous women are more likely to commit suicide than other Canadian women [7]. Suicide among Indigenous people is an issue of global concern and continues to devastate communities across Canada [61, 62]. Global evidence has shown that experiences of intimate partner violence among women are significantly associated with poor health, suicidal thoughts, prior attempts, and poor mental health [63]. Eynan and colleagues (2002) studied 330 homeless adults and found a high prevalence of suicidal ideation (61%). Furthermore, in their study 78% of female participants compared to 56% of male participants reported suicidal ideation, and 57% of the women had attempted suicide compared to 28% of the men [64, 65].

Substantial research has shown that suicide rates among Indigenous communities in Canada are of epidemic proportions. Despite significant investments, research investigating the determinants of suicidality within the Indigenous population and identifying promising interventions remains preliminary [66]. Indigenous scholars have raised concerns about using a homogenous approach to suicide interventions highlighting the diversity within the Indigenous population and variation in suicide rates. Research that examines intersectional meanings of identity, environment, Indigenous culture and community in relation to suicide is required [67]. Some scholars have recommended addressing Indigenous suicidality through the implementation of culturally relevant programming, which incorporates Indigenous worldviews, culture, ceremony, elders and spirituality [68]. Chandler has asserted that Indigenous communities with low suicide rates are marked by multiple community factors including: higher levels of self-determination on the community level, self-government, active attempts to restore traditional land title, preserve Indigenous language, culture and women’s roles in governance [68]. Individualistic approaches to addressing Indigenous suicide have failed continually and with an absence of promising interventions, Indigenous suicide research must be reoriented to focus on critical community level factors that foster cultural resurgence, reclamation and self-determination [67, 68].

It has been argued that mainstream definitions of homelessness do not adequately describe the meaning of Indigenous homelessness. For example, Thistle (2017) described Indigenous homelessness as not simply the lack of a structure for habitation but a disconnection and isolation from kin relations and relationships to the land as understood through Indigenous worldviews. Thistle calls for acknowledgment that racism and discrimination are entrenched in Canadian society and prevent Indigenous people from thriving. He asserts that homelessness must be culturally understood and that Indigenous worldviews and experiences must inform solutions [69]. The current study indicates that gender should be added to the contextualization of Indigenous homelessness, and must inform the development of interventions and supports. With abundant evidence that the unravelling of Indigenous cultural systems perpetuates Indigenous homelessness, it is clear that decolonizing approaches are essential, particularly for Indigenous women [1, 2, 61, 69].

Indigenous scholars have called for the development of Indigenous gender-based analytic approaches that account for the experiences of all members of communities including girls, women, trans and two spirit peoples [10]. Several studies have shown that LGBTQ2S^3^ young people are overrepresented among youth experiencing homelessness [69–72]. Research on LGBTQ2S homelessness is scarce yet it is important to acknowledge that Indigenous gendered experiences of homelessness, violence and mental health are not simply limited to binary gender experiences. Hunt (2015), describes the erasure of two-spirit people as part of colonial violence resulting from racialized gender hierarchies that has led to current conceptions of gender as binary in health research [10]. Although the current study did not collect data on two-spirit and other experiences of gender, this gap in research is important to acknowledge in the framing of gendered health disparities among Indigenous Canadians, particularly because Indigenous LGBTQ2S individuals are at risk for both violence and homelessness and yet are largely unrepresented in discussions, analyses and recommendations.

### Study limitations

Despite the strengths of our study design (e.g., large sample size, structured diagnostic interviews), several limitations should be considered. Our reliance on self-report may have introduced recall-bias and other sources of error. A study comparing self-report measures in the current study with corresponding administrative records supported the validity of information collected in our trials [73]. However, the validation did not include the primary variables included in the current study. Further, given that the participants were selected based on current mental disorder this may have further compromised some of their responses. Baseline interviews were conducted prior to randomization to a housing intervention, which may have introduced bias if participants believed that their responses might influence assignment. However, it is not clear what direction such bias might take. Gender was self-identified with the categories of male and female and did not allow for an examination of other sexual orientations. Indigenous ethnicity was also measured through self-report and categorized as “yes/no,” although participants were asked if they identify specifically with First Nations, Inuit, Métis, or status and non-status. This study was not adequately powered to investigate differences between these identity groups. As a result of a long history of mistreatment within the healthcare system, many Indigenous people choose not to identify as Aboriginal in survey instruments for fear of discrimination. It is unknown whether similar concerns may have suppressed disclosure of Indigenous ethnicity in our study. We combined samples between sites with very differing proportions of Indigenous people (15 vs 70%). These differences were expected based on prior knowledge of the Winnipeg and Vancouver contexts, but it is not clear whether there are meaningful differences between Indigenous participants in different sites. Further research is required to investigate and identify best practices for Indigenous people who are homeless and mentally ill, and their generalizability across settings. Housing First strategies have promise, particularly if they incorporate culturally-relevant services that account for trauma histories, gender, and structural violence. Our study was an exploratory investigation secondary to the main objective to examine housing stability post-randomization. Our multi-variable and univariate comparisons are preliminary and should be interpreted as hypothesis generating.

## Conclusions

Housing policies and interventions for Indigenous Canadians must incorporate evidence of distinct needs associated with gender. Indigenous people experience homelessness in the context of historical and contemporary colonization. Violence and ongoing trauma have particularly forceful effects on the wellness of Indigenous women, their families, and communities. Yuan et al. (2015) described a research agenda for violence against American Indian and Alaska native women, calling for participatory research to develop culturally appropriate, strengths based interventions [74]. Similarly, Canadian researchers Browne et al. (2016) have described an approach to developing Equity Oriented Services, emphasizing culturally-safe care, trauma- and violence-informed care, and contextually-tailored care, all operating in partnership with Indigenous people. Further research and Indigenous leadership are required to investigate how culturally safe, trauma informed care can be incorporated into existing housing policy and programming. The current study found that the repercussions of colonization were evident in distinct ways among Indigenous men and women who were living with significant psychological distress and homeless in Canada. Practices that are informed by these differences are essential to redressing the displacement of Indigenous women into homelessness, violence, and other forms of societal victimization.

## Data Availability

Data is stored at St. Michael’s Hospital in Toronto and is available to external investigators who sign a Data Sharing and Use Agreement that stipulates the responsibilities associated with transfer of datasets. Dr. Carol Adair at the University of Calgary is the data access coordinator. The corresponding author may be contacted for inquiries regarding data access.

## Abbreviations

AOR: Adjusted Odds Ratio
BC: British Columbia
CI: Confidence Interval
DTES: Downtown Eastside of Vancouver
ER: Emergency Room
EuroQol5D: Health Related Quality of Life Scale
HF: Housing First
HN: High Needs
IPV: Interpersonal violence;
LEC: Lived Experience Circle
LGBTQ2S: Lesbian, Gay, Bisexual, Trans, Queer, Two-Spirit
MCAS: Multnomah Community Ability Scale
MINI: Mini International Neuropsychiatric Interview: Maudsley Addiction Profile
PTSD: Post-Traumatic Stress Disorder
PWLE: Persons with lived experience
SRO: Single Room Occupancy Hotel
UOR: Unadjusted Odds Ratio

## Footnotes

1 The term ‘Indigenous’ will be used throughout this paper to collectively describe the Indigenous peoples of Canada, inclusive of those who identify as ‘Aboriginal’, or First Nations, Métis and Inuit. This term is used while also acknowledging the diversity of cultures, languages and traditions that exist among Indigenous Canadians.

2 **Housing First (HF)** originated from the Pathways model in New York City and involves building a portfolio of rental accommodations scattered in different neighbourhoods providing clients with meaningful choices in the location and setting of their residence. Clients are supported in their homes with Assertive Community Treatment or Intensive case management depending on their needs. HF has been shown to promote residential stability and community integration [35].

3 LGBTQ2S is used to refer to those who identify as Lesbian, Gay, Bisexual, Trans, Queer and Two-Spirit. Two Spirit is a term used in some Indigenous communities, encompassing sexual, gender, cultural and spiritual identity. Two-Spirit is reflective of complex understandings of gender in Indigenous cultures. There is a spiritual, cultural and historical component to being two-spirit originating from Indigenous cultural beliefs and practices pre-contact and it is important to acknowledge that this differs from identifying as LGBTQ+ Indigenous.
